# Supplementary material for: Dopamine and Serotonin Transporter Genes Regulation in Highly Sensitive Individuals during Stressful Conditions: A Focus on Genetics and Epigenetics
Source: Biomedicines. 2024 Sep 23;12(9):2149. doi: 10.3390/biomedicines12092149 (PMC11429336; doi:10.3390/biomedicines12092149)
Supplement: Supplementary file 1 [file biomedicines-12-02149-s001.zip › biomedicines-3183646-supplementary.pdf]

## Dopamine and Serotonin Transporter Genes Regulation in Highly Sensitive Individuals Under Stressful Conditions: Focus on Genetics and Epigenetics

**Table S1**. Characteristic of the study sample and subdivision of the groups based on HSP and PSS scores

| **SOCIO-DEMOGRAPHIC FEATURES** | | |
|---|---|---|
| **Gender (m;f)** | | 19; 85 |
| **Age at recruitment (mean ± SD)** | | 20.04 ± 1.77 |
| | | **N** |
| **HSP score** | Low (HSP<4) | 10 |
| | Medium (4<HSP<4.5) | 21 |
| | High (4.5<HSP) | 73 |
| **PSS score** | Low (PSS<13) | 18 |
| | Medium (14<PSS<26) | 55 |
| | High (27<PSS) | 31 |
| **HSPxPSS interaction** | Low | 17 |
| | Medium | 21 |
| | High | 66 |

**Table S2**. *DAT1* gene DNA methylation: groups' mean + SEM and Dunn's test P values for the individual comparisons. Highlighted in bold the significative results.

| PSS | | | | | | |
|---|---|---|---|---|---|---|
| **CpG site** | **Low** | **Moderate** | **High** | **Low vs Moderate** | **Low vs High** | **Moderate vs High** |
| 1 | 7.29 ± 0.48 | 7.40 ± 0.32 | 8.34 ± 0.35 | 0.8391 | 0.1254 | **0.0254** |
| 2 | 4.83 ± 0.53 | 4.53 ± 0.23 | 4.51 ± 0.29 | 0.9196 | 0.9274 | 0.9980 |
| 3 | 5.26 ± 0.32 | 4.90 ± 0.21 | 5.72 ± 0.42 | 0.4002 | 0.7730 | 0.1681 |
| 5 | 10.36 ± 0.79 | 10.74 ± 0.78 | 10.82 ± 0.91 | 0.4890 | 0.9711 | 0.4325 |
| 6 | 2.55 ± 0.32 | 2.66 ± 0.21 | 2.87 ± 0.36 | 0.7117 | 0.4611 | 0.6170 |
| 7 | 10.38 ± 1.01 | 11.79 ± 0.63 | 9.92 ± 0.36 | 0.2055 | 0.6695 | **0.0392** |
| Average | 6.73 ± 0.38 | 7.10 ± 0.22 | 7.13 ± 0.25 | 0.5458 | 0.5257 | 0.9166 |
| **HSP** | | | | | | |
| 1 | 6.81 ± 0.69 | 8.09 ± 0.58 | 7.62 ± 0.24 | 0.2645 | 0.2763 | 0.8167 |
| 2 | 4.53 ± 0.67 | 4.58 ± 0.44 | 4.63 ± 0.20 | 0.7051 | 0.9379 | 0.4547 |
| 3 | 5.68 ± 0.54 | 4.99 ± 0.38 | 5.25 ± 0.22 | 0.2591 | 0.4527 | 0.4473 |
| 5 | 8.76 ± 1.00 | 8.82 ± 0.70 | 11.45 ± 0.67 | 0.9564 | 0.1296 | **0.0326** |
| 6 | 2.88 ± 0.63 | 2.24 ± 0.21 | 2.85 ± 0.21 | 0.3937 | 0.9964 | 0.1442 |
| 7 | 10.38 ± 0.87 | 12.15 ± 1.001 | 10.86 ± 0.46 | 0.3805 | 0.8919 | 0.2075 |
| Average | 6.62 ± 0.41 | 6.87 ± 0.27 | 7.19 ± 0.19 | 0.7950 | 0.3558 | 0.3412 |
| **HSPxPSS** | | | | | | |
| 1 | 6.80 ± 0.60 | 7.59 ± 0.66 | 7.75 ± 0.25 | 0.6114 | 0.1786 | 0.4224 |
| 2 | 4.81 ± 0.52 | 4.36 ± 0.43 | 4.64 ± 0.20 | 0.3816 | 0.9777 | 0.2667 |
| 3 | 5.11 ± 0.37 | 4.89 ± 0.25 | 5.36 ± 0.26 | 0.6947 | 0.7659 | 0.4017 |
| 5 | 10.06 ± 0.80 | 8.29 ± 0.67 | 11.54 ± 0.72 | 0.1158 | 0.5716 | **0.0090** |
| 6 | 2.37 ± 0.32 | 2.40 ± 0.25 | 2.92 ± 0.23 | 0.7737 | 0.1718 | 0.2615 |
| 7 | 11.06 ± 0.80 | 11.73 ± 1.22 | 10.90 ± 0.45 | 0.7120 | 0.7232 | 0.3850 |
| Average | 6.85 ± 0.26 | 6.57 ± 0.37 | 7.29 ± 0.19 | 0.6688 | 0.3456 | 0.1120 |

**Table S3**. *SERT* gene DNA methylation: Dunn's test P values for the individual comparisons. Highlighted in bold the significative results.

| | | | | | | |
|---|---|---|---|---|---|---|
| **PSS** | | | | | | |
| **CpG site** | **Low** | **Moderate** | **High** | **Low vs Moderate** | **Low vs High** | **Moderate vs High** |
| 1 | 3.24 ± .018 | 2.85 ± 0.12 | 2.85 ± 0.14 | 0.0527 | **0.0445** | 0.7620 |
| 2 | 1.48 ± 0.10 | 1.33 ± 0.07 | 1.16 ± 0.08 | 0.0950 | 0.8660 | 0.0759 |
| 3 | 2.65 ± 1.18 | 2.55 ± 0.15 | 2.70 ± 0.13 | 0.2706 | 0.7003 | 0.0612 |
| 4 | 3.21 ± 0.41 | 2.95 ± 0.26 | 3.49 ± 0.48 | 0.4783 | 0.8535 | 0.2808 |
| 5 | 2.95 ± 0.67 | 2.21 ± 0.23 | 2.21 ± 0.12 | 0.0629 | 0.5109 | 0.1630 |
| 6 | 2.20 ± 0.36 | 0.00 ± 0.27 | 2.10 ± 0.21 | 0.6732 | 0.6804 | 0.3221 |
| Average | 2.64 ± 0.16 | 2.32 ± 0.08 | 2.49 ± 0.12 | 0.0593 | 0.4367 | 0.2095 |
| **HSP** | | | | | | |
| 1 | 3.02 ± 0.20 | 2.89 ± 0.18 | 2.91 ± 0.10 | 0.3956 | 0.4316 | 0.8067 |
| 2 | 1.36 ± 0.15 | 1.31 ± 0.09 | 1.43 ± 0.06 | 0.8473 | 0.9915 | 0.7456 |
| 3 | 2.41 ± 0.19 | 2.52 ± 0.18 | 2.67 ± 0.12 | 0.8436 | 0.6714 | 0.7735 |
| 4 | 3.26 ± 0.44 | 2.73 ± 0.34 | 3.28 ± 0.27 | 0.1441 | 0.5000 | 0.1604 |
| 5 | 1.96 ± 0.31 | 1.99 ± 0.18 | 2.50 ± 0.23 | 0.9258 | 0.3913 | 0.1486 |
| 6 | 1.77 ± 1.19 | 2.64 ± 0.66 | 1.93 ± 0.12 | 0.9184 | 0.9617 | 0.9236 |
| Average | 2.29 ± 0.11 | 2.33 ± 0.13 | 2.47 ± 0.08 | 0.8524 | 0.5973 | 0.2924 |
| **HSPxPSS** | | | | | | |
| 1 | 3.14 ± 0.19 | 2.87 ± 0.15 | 2.88 ± 0.11 | 0.1704 | 0.1106 | 0.9670 |
| 2 | 1.41 ± 0.08 | 1.39 ± 0.12 | 1.39 ± 0.06 | 0.8691 | 0.4747 | 0.5640 |
| 3 | 2.52 ± 0.14 | 2.61 ± 0.22 | 2.64 ± 0.13 | 0.8123 | 0.9496 | 0.8090 |
| 4 | 3.01 ± 0.33 | 3.11 ± 0.43 | 3.22 ± 0.29 | 0.6090 | 0.8429 | 0.6482 |
| 5 | 2.19 ± 0.24 | 2.61 ± 0.58 | 2.32 ± 0.19 | 0.9255 | 0.9677 | 0.8675 |
| 6 | 2.19 ± 0.38 | 2.44 ± 0.63 | 1.91 ± 0.12 | 0.5884 | 0.6824 | 0.7991 |
| Average | 2.40 ± 0.11 | 2.49 ± 0.16 | 2.41 ± 0.08 | 0.9818 | 0.8814 | 0.8444 |

**Table S4**. Genotypes and alleles distribution of *DAT1* VNTR and *SERT* 5-HTTLPR between the groups based on subjects' PSS score. Data are reported both in number and percentage for each genotype and allele. Data were analysed with the Chi-square test. Chi-square values, degrees of freedom, and P values are reported on the right. Highlighted in bold the significative results.

| | Perceived Stress Scale – PSS (N) | | | | |
|---|---|---|---|---|---|
| **Genotype/ Allele** | **PSS < 13 (10)** | **14 < PSS < 26 (21)** | **PSS > 27 (73)** | **Chi-square, df** | **P value** |
| ***SERT* (5-HTTLPR)** | | **genotypes/alleles (n, %)** | | | |
| LL | 6 (5.77) | 29 (27.88) | 17 (16.35) | | |
| LS | 8 (7.69) | 11 (10.58) | 8 (7.69) | 4.89, 4 | 0.2982 |
| SS | 4 (3.85) | 15 (14.42) | 6 (5.77) | | |
| L | 20 (9.62) | 69 (33.17) | 42 (20.19) | 1.45, 2 | 0.4826 |
| S | 16 (7.69) | 41 (19.71) | 20 (9.62) | | |
| ***DAT1* VNTR** | | **genotypes/alleles (n, %)** | | | |
| 7/7 | 0 (0) | 2 (1.92) | 1 (0.96) | | |
| 7/8 | 3 (2.88) | 3 (2.88) | 1 (0.96) | | |
| 8/8 | 3 (2.88) | 4 (3.85) | 2 (1.92) | | |
| 8/9 | 0 (0) | 3 (2.88) | 0 (0) | 16.66, 12 | 0.1629 |
| 9/9 | 2 (1.92) | 12 (11.54) | 3 (2.88) | | |
| 9/10 | 5 (4.81) | 21 (20.19) | 8 (7.69) | | |
| 10/10 | 5 (4.81) | 10 (9.62) | 16 (15.38) | | |
| 7 | 3 (1.44) | 7 (3.37) | 3 (1.44) | | |
| 8 | 9 (4.33) | 14 (6.73) | 5 (2.40) | 18.17, 6 | **0.0058** |
| 9 | 9 (4.33) | 48 (23.08) | 14 (6.73) | | |
| 10 | 15 (7.21) | 41 (19.71) | 40 (19.23) | | |

**Table S5**. Genotypes and alleles distribution of *DAT1* VNTR and *SERT* 5-HTTLPR between the groups based on subjects' HSP score. Data are reported both in number and percentage for each genotype and allele. Data were analysed with the Chi-square test. Chi-square values, degrees of freedom, and P values are reported on the right.

| | Highly Sensitive Person test – HSP (N) | | | | |
|---|---|---|---|---|---|
| **Genotype/ Allele** | **HSP < 4 (18)** | **4 ≤ HSP ≤ 4.5 (55)** | **HSP > 4.5 (31)** | **Chi-square, df** | **P value** |
| ***SERT* (5-HTTLPR)** | | **genotypes/alleles (n, %)** | | | |
| LL | 4 (3.85) | 9 (8.65) | 39 (97.50) | | |
| LS | 2 (1.92) | 7 (6.73) | 18 (17.31) | 2.41, 4 | 0.6614 |
| SS | 4 (3.85) | 5 (4.81) | 16 (15.38) | | |
| L | 10 (4.81) | 25 (12.02) | 96 (46.15) | 2.14, 2 | 0.3427 |
| S | 10 (4.81) | 17 (8.17) | 50 (24.04) | | |
| ***DAT1* VNTR** | | **genotypes/alleles (n, %)** | | | |
| 7/7 | 1 (0.96) | 0 (0) | 2 (1.92) | | |
| 7/8 | 1 (0.96) | 2 (1.92) | 4 (3.85) | | |
| 8/8 | 0 (0) | 1 (0.96) | 8 (7.69) | | |
| 8/9 | 0 (0) | 1 (0.96) | 2 (1.92) | 10.46, 12 | 0.5758 |
| 9/9 | 3 (2.88) | 2 (1.92) | 12 (11.54) | | |
| 9/10 | 4 (3.85) | 9 (8.65) | 21 (20.19) | | |
| 10/10 | 1 (0.96) | 6 (5.77) | 24 (23.08) | | |
| 7 | 3 (1.44) | 2 (0.96) | 8 (3.85) | | |
| 8 | 1 (0.48) | 5 (2.40) | 22 (10.58) | 7.11, 6 | 0.3112 |
| 9 | 10 (4.81) | 14 (6.73) | 47 (22.60) | | |
| 10 | 6 (2.88) | 21 (10.10) | 69 (33.17) | | |

**Table S6**. Genotypes and alleles distribution of *DAT1* VNTR and *SERT* 5-HTTLPR between the groups based on subjects' HSPxPSS score. Data are reported both in number and percentage for each genotype and allele. Data were analysed with the Chi-square test. Chi-square values, degrees of freedom, and P values are reported on the right.

| | | **HSPxPSS (N)** | | | |
|---|---|---|---|---|---|
| **Genotype/ Allele** | **Low-risk (17)** | **Medium-risk (21)** | **High-risk (66)** | **Chi-square, df** | **P value** |
| ***SERT* (5-HTTLPR)** | | **genotypes/alleles (n, %)** | | | |
| LL | 8 (7.69) | 6 (5.77) | 38 (36.54) | | |
| LS | 4 (3.85) | 9 (8.65) | 14 (13.46) | 6.24, 4 | 0.1819 |
| SS | 5 (4.81) | 6 (5.77) | 14 (13.46) | | |
| L | 20 (9.62) | 21 (10.10) | 90 (43.27) | 4.81, 2 | 0.0899 |
| S | 14 (6.73) | 21 (10.10) | 42 (20.19) | | |
| ***DAT1* VNTR** | | **genotypes/alleles (n, %)** | | | |
| 7/7 | 1 (0.96) | 0 (0) | 2 (1.92) | | |
| 7/8 | 3 (2.88) | 1 (0.96) | 3 (2.88) | | |
| 8/8 | 0 (0) | 4 (3.85) | 5 (4.81) | | |
| 8/9 | 0 (0) | 1 (0.96) | 2 (1.92) | 14.74, 12 | 0.2562 |
| 9/9 | 4 (3.85) | 1 (0.96) | 12 (11.54) | | |
| 9/10 | 6 (5.77) | 9 (8.65) | 19 (18.27) | | |
| 10/10 | 3 (2.88) | 5 (4.81) | 23 (22.12) | | |
| 7 | 5 (2.40) | 1 (0.48) | 7 (3.37) | | |
| 8 | 3 (1.44) | 10 (4.81) | 15 (7.21) | 11.42, 6 | 0.0761 |
| 9 | 14 (6.73) | 12 (5.77) | 45 (21.63) | | |
| 10 | 12 (5.77) | 19 (9.13) | 65 (31.25) | | |

**Table S7**. miRNAs expression: Dunn's test P values for the individual comparisons. Highlighted in bold the significative results.

| | | miR-132 | miR-491 | miR-135 | miR-16-5p |
|---|---|---|---|---|---|
| **PSS** | Low vs Medium | 0.5910 | 0.8379 | 0.3866 | 0.8312 |
| | Low vs High | 0.5167 | 0.4923 | 0.1420 | 0.7114 |
| | Medium vs High | 0.8137 | 0.2754 | **0.0051** | 0.8174 |
| | | | | | |
| **HSP** | Low vs Medium | 0.3886 | 0.1265 | 0.3471 | 0.2338 |
| | Low vs High | 0.5376 | 0.5740 | 0.1293 | 0.5623 |
| | Medium vs High | 0.6203 | 0.1473 | 0.5404 | 0.2839 |
| | | | | | |
| **HSPxPSS** | Low vs Medium | 0.1814 | 0.2187 | 0.7191 | 0.5854 |
| | Low vs High | 0.2226 | 0.4270 | 0.2184 | 0.5628 |
| | Medium vs High | 0.6874 | **0.0196** | 0.0838 | 0.1978 |

**Table S8**. miRNAs expression correlation considering the PSS scores. In the lower part of the table are reported the Spearman's R values; in the upper part of the table are reported the p values. Highlighted in bold the significative results.

| | **miR-132 (DAT 3'UTR)** | **miR-135 (SERT2)** | **miR-491 (DAT1 VNTR)** | **miR-16-5p** |
|---|---|---|---|---|
| **miR-132 (DAT 3'UTR)** | | **2.5153e-005** | 0.1252 | 0.3479 |
| **miR-135 (SERT2)** | 0.4376 | | **0.0491** | 0.5368 |
| **miR-491 (DAT1 VNTR)** | 0.1666 | 0.2142 | | 0.7008 |
| **miR-16-5p** | 0.1001 | 0.0667 | -0.0413 | |

**Table S9**. miRNAs expression correlation considering the HSP scores. In the lower part of the table are reported the Spearman's R values; in the upper part of the table are reported the p values. Highlighted in bold the significative results.

| | **miR-132 (DAT 3'UTR)** | **miR-135 (SERT2)** | **miR-491 (DAT1 VNTR)** | **miR-16-5p** |
|---|---|---|---|---|
| **miR-132 (DAT 3'UTR)** | | **1.9799e-005** | 0.2007 | 0.5486 |
| **miR-135 (SERT2)** | 0.4379 | | **0.0371** | 0.6670 |
| **miR-491 (DAT1 VNTR)** | 0.1647 | 0.2632 | | 0.2311 |
| **miR-16-5p** | 0.0637 | 0.0458 | -0.1543 | |

**Table S10**. miRNAs expression correlation considering the HSPxPSS scores. In the lower part of the table are reported the Spearman's R values; in the upper part of the table are reported the p values. Highlighted in bold the significative results.

| | miR-132 (DAT 3'UTR) | miR-135 (SERT2) | miR-491 (DAT1 VNTR) | miR-16-5p |
|---|---|---|---|---|
| **miR-132 (DAT 3'UTR)** | | **2.7854e-004** | 0.1055 | 0.5399 |
| **miR-135 (SERT2)** | 0.3868 | | 0.3153 | 0.6315 |
| **miR-491 (DAT1 VNTR)** | -0.2026 | 0.1255 | | 0.2799 |
| **miR-16-5p** | 0.0662 | 0.0525 | -0.1329 | |
